# The RCSB PDB “Molecule of the Month”: Inspiring a Molecular View of Biology

**DOI:** 10.1371/journal.pbio.1002140

**Published:** 2015-05-05

**Authors:** David S. Goodsell, Shuchismita Dutta, Christine Zardecki, Maria Voigt, Helen M. Berman, Stephen K. Burley

**Affiliations:** 1 RCSB Protein Data Bank; 2 Department of Integrative Structural and Computational Biology, The Scripps Research Institute, La Jolla, California, United States of America; 3 Center for Integrative Proteomics Research and Department of Chemistry and Chemical Biology, Rutgers, The State University of New Jersey, New Brunswick, New Jersey, United States of America; 4 BioMaPS Institute for Quantitative Biology and Cancer Institute of New Jersey, Rutgers, The State University of New Jersey, New Brunswick, New Jersey, United States of America; 5 San Diego Supercomputer Center and Skaggs School of Pharmacological Sciences, University of California, San Diego, San Diego, California, United States of America

## Abstract

The Research Collaboratory for Structural Bioinformatics (RCSB) Molecule of the Month series provides a curated introduction to the 3-D biomolecular structures available in the Protein Data Bank archive and the tools that are available at the RCSB website for accessing and exploring them. A variety of educational materials, such as articles, videos, posters, hands-on activities, lesson plans, and curricula, build on this series for use in a variety of educational settings as a general introduction to key topics, such as enzyme action, protein synthesis, and viruses. The series and associated educational materials are freely available at www.rcsb.org.

## Introduction

Three-dimensional (3-D) structures of biological macromolecules are important because “function follows form” in biology. Knowledge of the shape, structure, and interactions of biological macromolecules (proteins, nucleic acids, and carbohydrates) helps us understand and “see” biology at the molecular level in atomic detail [1]. Changes in shape, chemical properties, and assemblages of biomolecules are critical to understanding disease processes and in discovering and developing targeted treatments for diseases. Since 2000, a monthly feature at the Research Collaboratory for Structural Bioinformatics Protein Data Bank (RCSB PDB) [2], called “Molecule of the Month” (MotM), has highlighted structures and functions of selected biological macromolecules and helped explain their importance in biology and medicine. Today, more than 180 of these monthly features provide users with a curated sampling of the Protein Data Bank (PDB).

Interactive tutorials describing biomolecular subjects were pioneered by Eric Martz [3], initially using Chime and Protein Explorer and more recently using JMol/JSMol. Concurrent with development of the Molecule of the Month at the RCSB PDB, several projects have developed related resources that allow users to view and explore PDB structures, including the InterPro “Protein Focus” (formerly known as “Protein of the Month”) and "Quips" [4], both of which come from the European Bioinformatics Institute (EMBL-EBI), and Proteopedia [5] from the Weizmann Institute, which uses a crowdsourcing approach based on Wikipedia.

Established in 1971 as the first open access digital resource in biology, the PDB [6] provides free, global access to more than 100,000 experimentally determined, 3-D, atomic structures of biological macromolecules. These data are widely used by structural biologists and other scientists working in basic and applied sciences. The same data are also made available to students, educators, and general audiences to promote understanding and discovery of new science. The PDB archive is maintained by the worldwide PDB organization (wwPDB), which today encompasses regional data centers in the United States (RCSB PDB; http://www.rcsb.org) at Rutgers University and University of California, San Diego; the Biological Magnetic Resonance Data Bank at University of Wisconsin, Madison; and centers in Europe (Protein Data Bank in Europe, PDBe; http://pdbe.org) and Japan (Protein Data Bank in Japan, PDBj; http://pdbj.org) [6]. The wwPDB data centers collaboratively manage data deposition and validation. Thereafter, each data center provides online access to contents of the PDB archive. The primary data distributed by all centers is the same, but each center offers different views, tools, and resources for accessing and using the information. The RCSB PDB [7] offers both expert (http://www.rcsb.org) and introductory/educational portals (http://www.rcsb.org/pdb-101) to the PDB archive [8].

The PDB-101 educational portal allows non-expert users to explore biology through a structural lens and learn how to use and understand PDB data. This resource presents a wide range of educational materials, such as articles, videos, posters, hands-on activities, lesson plans, and curricula (Box 1). Since 2000, the MotM has profiled molecular structures, together with RCSB PDB tools to explore them. Herein, we describe the conceptual design of these features, their use in research and education, and their ability to bring a structural view of biology and medicine to a general audience.

**Box 1. PDB-101 at a Glance**

PDB-101 (http://www.rcsb.org/pdb-101) provides an introduction to key structural biology topics and access to relevant biomolecular structures. Monthly MotM features serve as a bridge between traditional teaching materials and the primary scientific data, which is freely available without restrictions on usage from the PDB archive.

**Key components**

- Molecule of the Month—Short, illustrated features on selected molecules from the archive provide a sampling of the PDB holdings.
- Structural View of Biology Browser—MotM features are organized by theme for exploration by all users.
- Educational Resources—Various materials, including posters, articles, hands-on activities, images, and animations, are available for educational use.
- Video Challenge—Instructions, rules, resources, and information related to a competition for high school students are available here.
- Curricula—Curricula have been developed through collaborations and participation of scientists, curriculum design experts, educators, clinicians, and local teachers.

## Design of the Molecule of the Month

**Fig 1:**
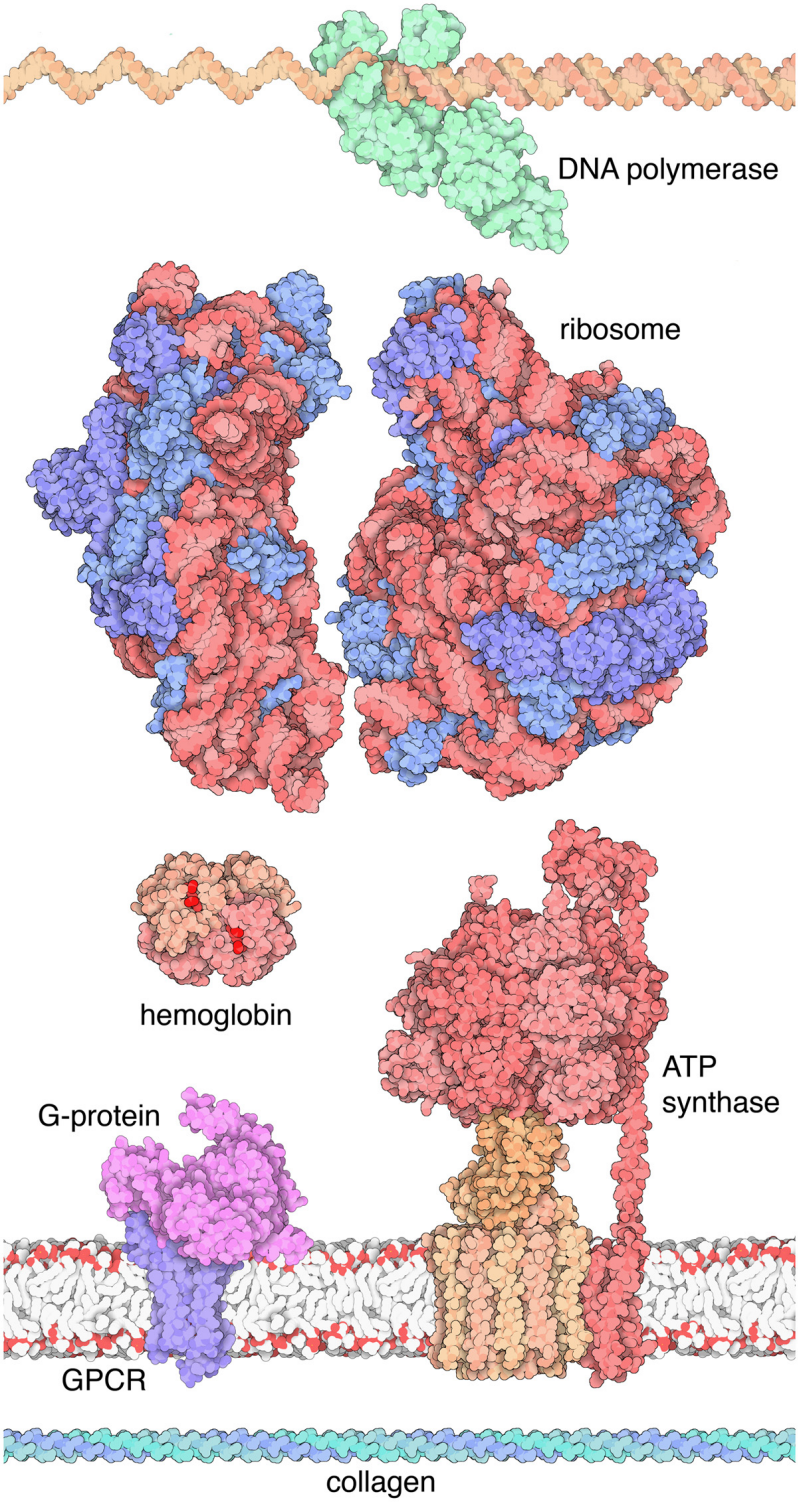
Molecules highlighted in the MotM features. As of the end of 2014, 180 different molecules have been presented in Molecule of the Month features. Topics are selected to promote understanding of biomolecular structure and function and connections to human health and disease as well as biotechnology. Some of the more popular subjects are depicted herein. From top to bottom: DNA polymerase [16], ribosome [17,18], hemoglobin [19], ATP synthase [20,21], a G-protein bound to a G-protein-coupled receptor (GPCR) [22], and the collagen triple helix [23].

**Fig 2:**
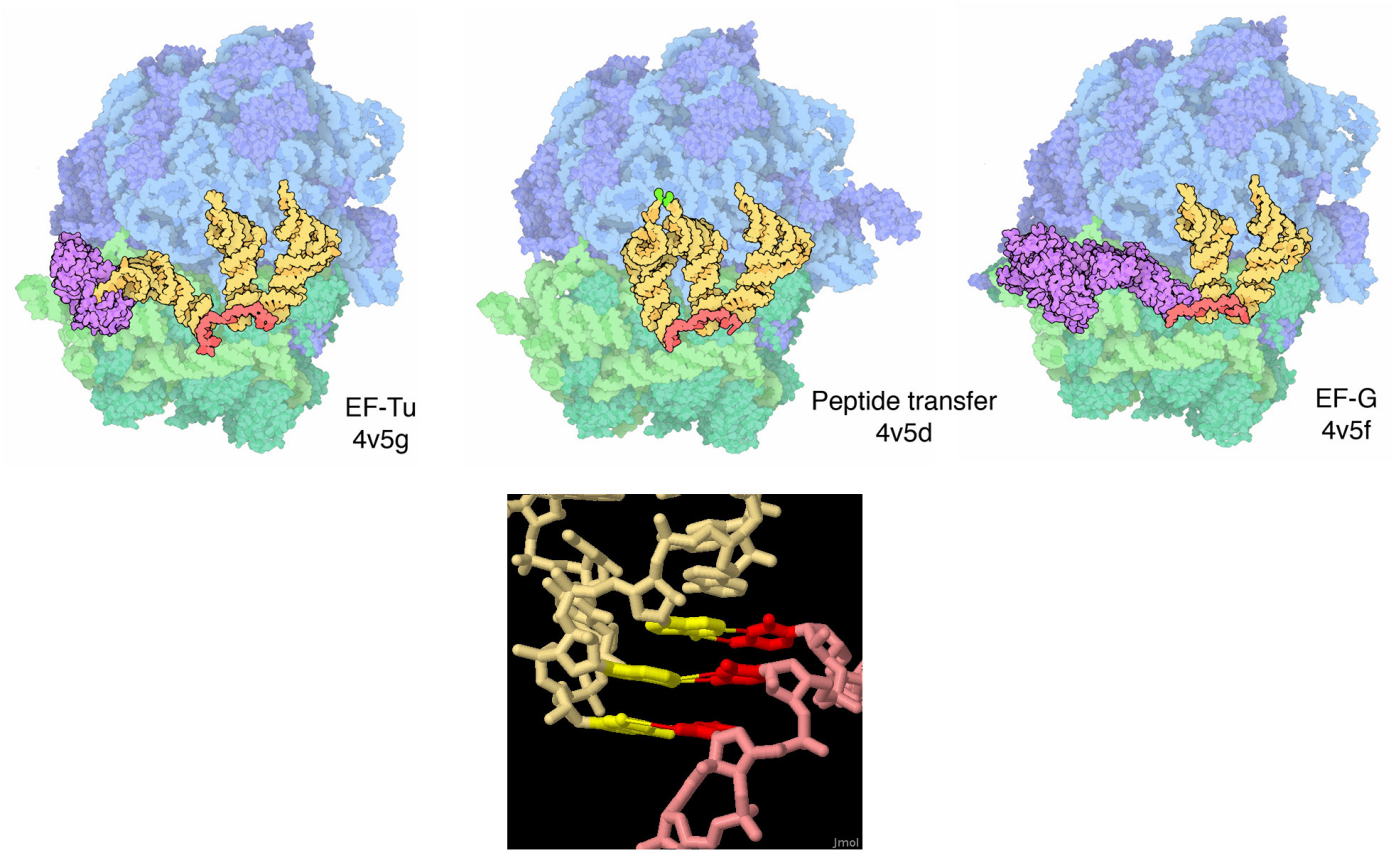
Images from MotM features. For the 2010 column on the ribosome, structures were chosen to exemplify discrete mechanistic steps in protein synthesis, including the elongation phase (top) [24–26]. The style of the illustration highlights major molecular players: ribosomal subunits in blue (large) and green (small), transfer RNA in yellow, messenger RNA in red, and elongation factors in purple. The non-photorealistic style is designed to focus attention on overall shape, size, and molecular interactions, while still giving a sense of the component atoms. An interactive JSmol was also included in the "Exploring the Structure" section of the column, allowing users to explore firsthand the codon-anticodon interaction (bottom). This illustration shows a close-up of the "decoding center" of the ribosome.

MotM features introduce 3-D structures of proteins, nucleic acids, and carbohydrates in the context of basic and applied biological and medical research. Themes for the features are selected from topics of general interest (Box 2, Fig 1), including molecules related to human health and disease (for example, hemoglobin and collagen), biological machines (ribosomes), and current events (Ebola virus, ricin, anthrax, and HIV/AIDS). Non-technical text explains the overall shapes of relevant biomolecules and highlights structural features important for their functions while illustrations highlight molecular structural features described in the text (Fig 2). Accompanying interactive JSmol views present curated explorations of one or more relevant structures allowing users to view them in 3-D. (JSmol is an open-source HTML5 viewer for chemical structures, available at http://wiki.jmol.org.)

**Box 2. Learning about Biology from MotM Features**

Topics presented in MotM features can be organized by user educational background.

**Elementary and Middle School**

- Biological Information: The primary feature on DNA highlights the double helical structure of B-DNA and the nature of Watson-Crick base pairing. Other features explain how DNA is read, duplicated, regulated, and repaired.
- Viruses: Many features on well-known viruses, such as Ebola virus, HIV, influenza, and rhinovirus help to show the science behind the news.

**High School (General Biology)**

- Mechanism of Protein Synthesis: A feature on ribosomes explains the multi-step process of protein synthesis. Other features describe supporting players, such as the transfer RNAs and elongation factors.
- Molecular Recognition: An introductory feature on hemoglobin presents an example of molecular recognition, highlighting protein motions that regulate oxygen binding in the lung and release in the blood stream.

**High School (AP Biology), Undergraduate, and Beyond**

- Enzyme Action: Features describe various enzymes commonly used in research, including carbonic anhydrase and catalase, revealing how structure underpins enzymatic activity.
- Molecular Basis of Disease: Many features describe molecules involved in cancer, explaining the connection between human diseases and gene mutations affecting protein structure and function.
- Biotechnology and Nanotechnology: Several features highlight molecular structures designed by scientists, such as artificial DNA lattices and protein cages, revealing how understanding of molecular structure and function can be put to practical use.

Links to featured PDB structures allow users to access specific RCSB PDB pages containing detailed information about the structure. In addition, links to abridged versions of the structure pages provide access to short descriptions of its biological function, an interactive JSmol 3-D interactive viewer, and sequences of protein and nucleic acid polymers of the molecules. Related PDB structures are also provided as starting points for further explorations, giving non-experts access to a curated subset of PDB structures to increase familiarity with visualization and help them take advantage of more complex resources, including more powerful visualization tools, available from the RCSB PDB. Users can access the features by topic (such as "Health and Disease" or "Biotechnology and Nanotechnology") or an alphabetical index of categories.

MotM features are widely used by diverse audiences (Box 3). Examples of educational programs, projects, and resources developed based on or impacted by our efforts are described in the following sections.

**Box 3. Usage of the Molecule of the Month**

Access and download statistics of individual monthly features over a period of time (e.g., approximately 18,000 unique visits for the feature on hemoglobin during the first nine months of 2014) document their popularity. Interestingly, MotM features are not only accessed immediately upon publication, but continue to be used many years later. An online survey of PDB-101 users, conducted in 2014, revealed that 74% of the approximately 700 respondents used one or more MotM features, with 49% downloading images for their own use, 46% using the interactive JSmol viewer, 40% viewing related structures, and 33% using our suggestions for exploration of related topics, provided at the end of each feature. While this survey is not exhaustive, it provides self-reported measures of which feature components are being used. Complementary metrics available from Google Analytics (http://www.google.com/analytics) documented over 350,000 sessions that accessed MotM pages between August 1, 2014, and January 18, 2015. These sessions originated from various search engines, links from scientific databases worldwide, and resources such as Foldit (http://fold.it/portal). MotM content is also reproduced with formal written permission by textbook authors, editors, and journalists (25 requests were processed in 2014).

### Molecule of the Month In Student Research

Two projects initiated at the Center for BioMolecular Modeling at the Milwaukee School of Engineering (CBM MSOE) illustrate how student research projects incorporate the MotM. The Connecting Researchers, Educators and Students (CREST) program (http://cbm.msoe.edu/stupro/crest) pairs local university researchers and educators with undergraduate students to work together on a research topic. MotM features have been assigned as foundational reading material for several of the CREST projects and have been incorporated into their posters, videos, tutorials, and scientific articles for use in undergraduate education.

In another CBM program, teachers from high schools nationwide learn about biological molecules by visualizing protein structures using MotM features. The teachers then lead a team of students to work with a local scientist on a research topic in a program called Students Modeling a Research Topic (SMART) teams. The teams model biomolecular structures based on available literature under the scientist’s guidance, often using MotM features for background research. Physical models created by these SMART teams provide useful “tools” for discussing the topic and designing new experiments [9,10]. The teams often present their work and models at local, regional, or national meetings organized by professional societies (e.g., ASBMB), giving students in these teams a taste of research experience.

### Informing and Challenging the Next Generation of Citizens and Scientists

**Fig 3:**
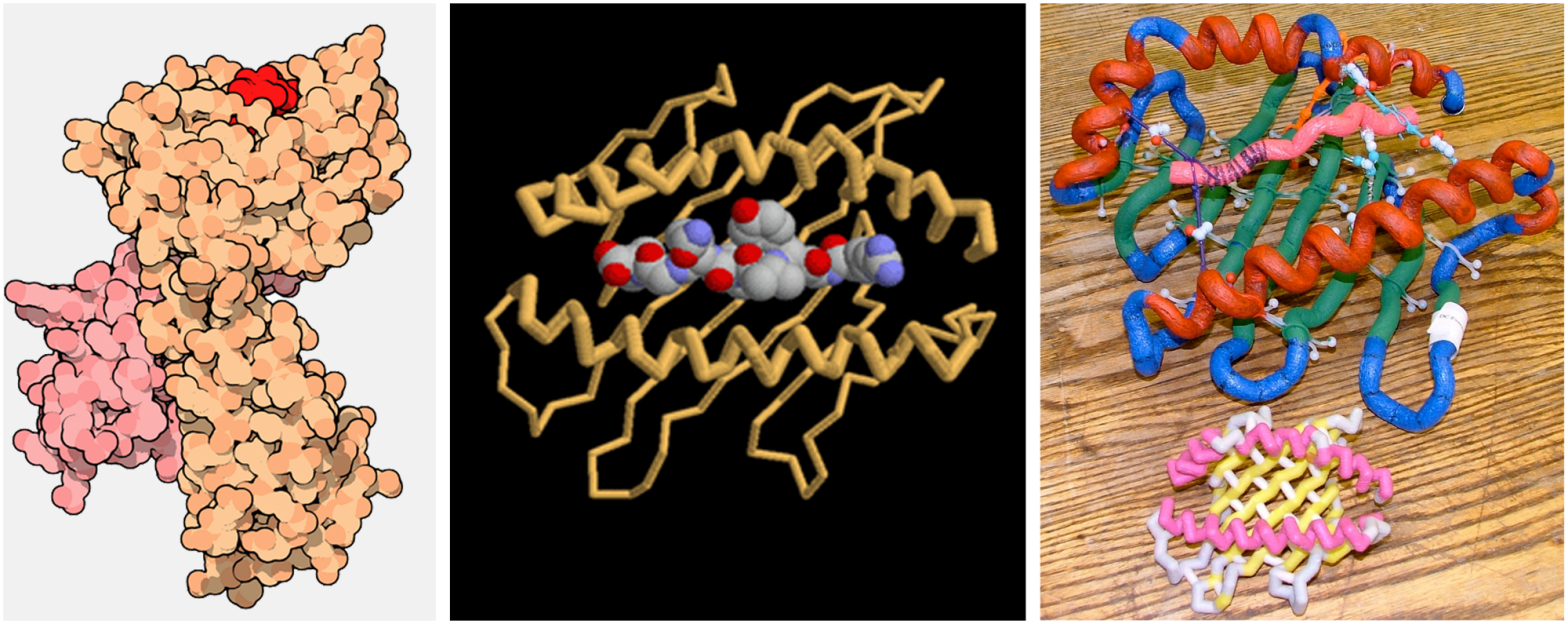
Protein modeling at the Science Olympiad. The Protein Modeling event uses MotM features to provide a scientific foundation. An example of how the models of the Major Histocompatibility Complex [27] are built in the competition is shown. Illustrations (left panel) and descriptions introduce the competition’s topic and the section on Exploring the Structure (central panel) and JSmol interactive views show details of the functionally important regions of the protein model. Students visualize the assigned molecules in the CBM MSOE environment to guide their model building. An example of student-built toober model is shown (top model in the right panel) along with a 3-D printed model of the same protein (lower model in the right panel, lacking the bound peptide) that event officials use for judging the model. Images in the right panel were kindly provided by Tim Herman, with permission.

The high school student protein modeling event in the Science Olympiad (http://soinc.org) [11], a large, nationwide scientific challenge, was originally developed by CBM MSOE and is now being operated jointly by RCSB PDB and CBM MSOE (Fig 3). To participate, students are required to read about a specific theme, build 3-D molecular models of proteins, and answer exam questions about the biology of the modeled molecular structures [12]. Links to specially prepared or extant MotM features provide the necessary background information for participants and serve as the source for many of the exam questions. Annually, 5,000–7,000 teams (with two to three students per team) participate throughout the US. Competitions are conducted at regional and state levels in late fall through early spring, leading up to the national-level competitions in May. Preparation for the competitions motivates students, teachers, coaches, and judges to read MotM features relevant to the competition.

Other competitions and extracurricular scientific events use MotM features as foundational materials, as well. For example, the RCSB PDB video challenge for high school students, started in 2014, challenged students to create two-minute videos highlighting aspects of the structural biology of HIV [13]. Challenge contributors with diverse interests cited the MotM features as resources used for creating their videos. Another example is noted in RCSB PDB’s "Education Corner" newsletter: the Protein Modeling Challenge at Stony Brook University, which pointed students to MotM features for background research on Ras proteins as molecular switches [14].

### Molecule of the Month In the Classroom

**Fig 4:**
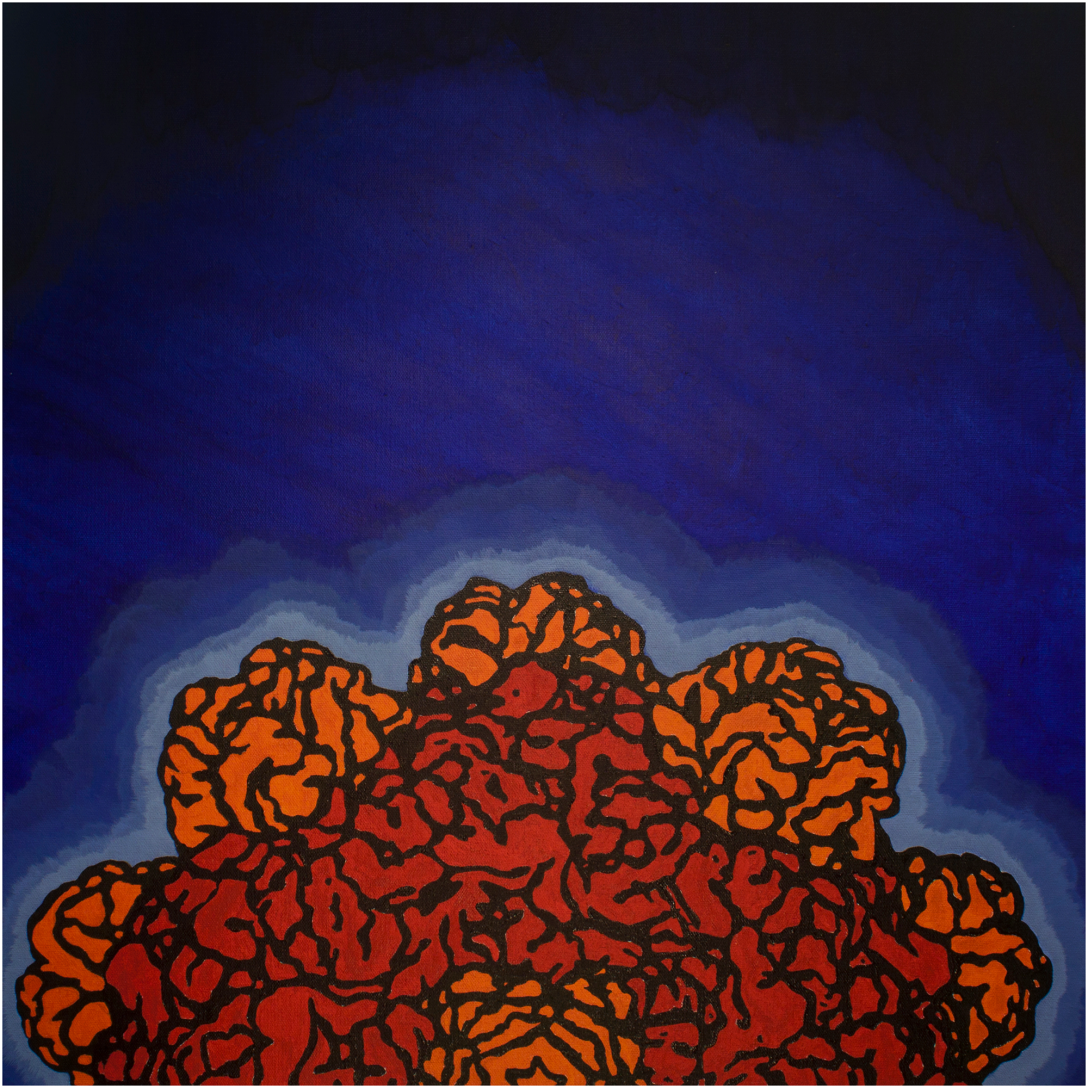
Molecular machines at High Tech High. Parag Chowdhury and his students at High Tech High in North County San Diego, California, combined art and science to explore molecular machines. Using the RCSB PDB MotM features as starting points, they created original paintings of biomolecules and wrote one-page descriptions of the role played by each biomolecule in the cell. They then combined all of this creative work into a book, *Molecular Machines*: *How Are We Assembled*?, currently available through the on-demand publisher Blurb. The painting of a bacterial virus or bacteriophage [28], shown above, was used on the cover of the book, and was kindly provided by Parag Chowdhury with permission.

**Fig 5:**
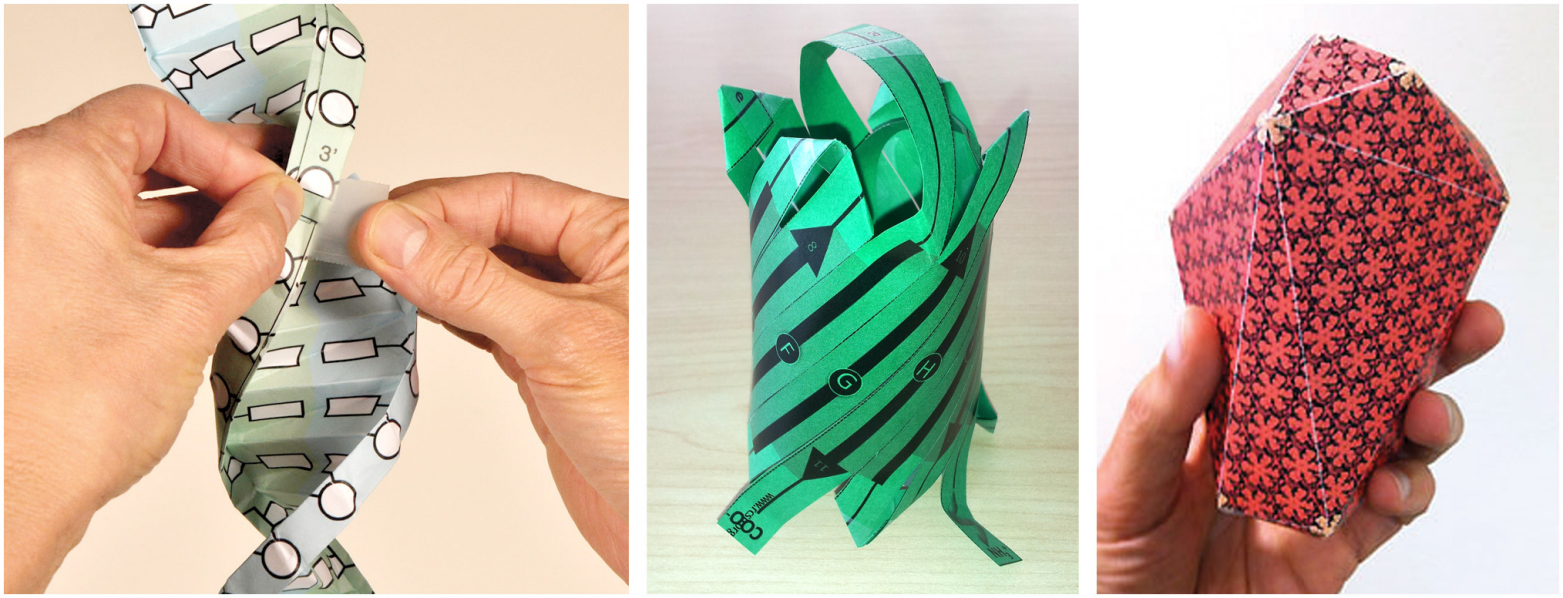
Molecular origami. Templates for paper models of selected MotM-featured structures present the opportunity to make low cost and easily accessible physical models. Integrating these models with the MotM features provides new perspectives on these molecules. Examples of a few paper models include: DNA double helix (left panel), green fluorescent protein (center panel) [29], and HIV capsid (right panel) [30]. These and other paper models and associated MotM features are available for download from http://www.pdb.org/pdb/101/static101.do?p=education_discussion/educational_resources/index.html.

We have received many anecdotal reports from educators using MotM features in manifold and creative ways. Some have assigned different topics to each student for presentation in their classrooms, using relevant MotM features as starting points for further study. One educator combined art with science, creating original paintings of molecules using proteins highlighted in MotM features as sources of inspiration (Fig 4). This project is particularly exciting, as the students are drawing directly from primary scientific data. Professional curriculum developers include links to MotM features (see, for instance, [15] and http://www.hhmi.org/biointeractive/how-do-fibers-form). As part of its outreach and educational mission, the RCSB PDB has developed short lesson plans on topics, such as double helical DNA, green fluorescent protein, and the HIV capsid, in which relevant MotM features are combined with hands-on activities (Fig 5). In addition to engaging students, these lesson plans are used to train teachers at local and national workshops. Scientists at all levels (undergraduate, graduate, postdoctoral, and faculty) also use these lesson plans and materials in specialist courses, such as in the Applied Bioinformatics Course at Peking University (since 2000) and the annual Interdisciplinary Quantitative Biology Boot Camp at Rutgers University (since 2014).

**Fig 6:**
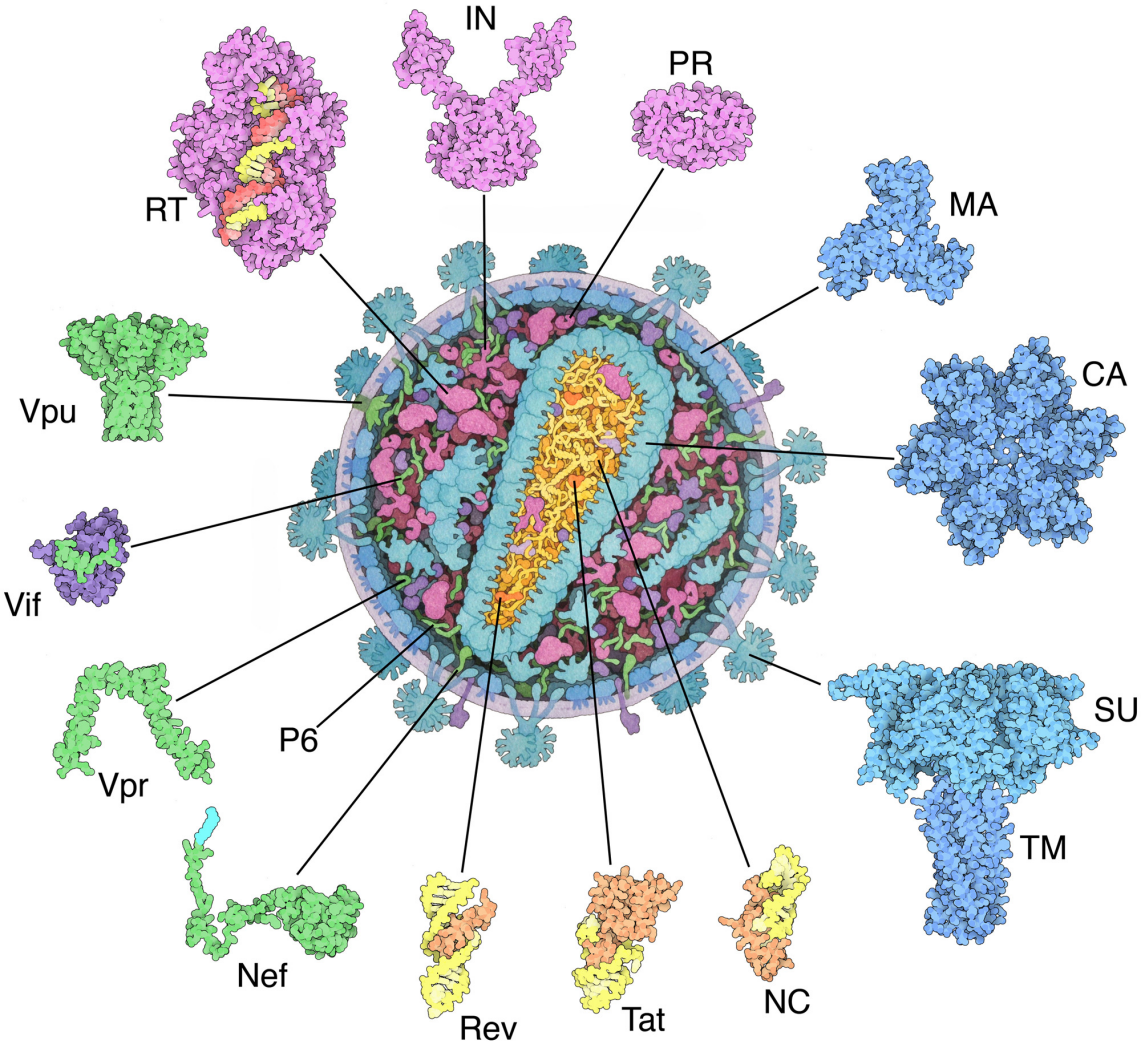
HIV/AIDS curriculum. Selected MotM features and educational materials developed at the RCSB PDB, such as this image of the anatomy of HIV and the proteins encoded by the HIV genome, are combined with other public resources in a curriculum that explores immunity and our defenses against HIV. The modular curriculum includes three main components: (i) review of the structure and function of biological molecules; (ii) introduction to the human immune system; and (iii) module on HIV/AIDS biology, treatments, and global impact. Two versions of the curricula are available for use in general biology and AP biology classrooms together with suggestions for assessing student learning. In addition to curricular material, pre- and post-tests are included. The curricula conform to current science teaching standards and core curricula and are available at http://education.rcsb.org/curriculum.

Our recent survey of PDB-101 users documented that, today, a majority of our users come from colleges, universities, and research institutes. Only 8% of respondents identified themselves as being from K–12 institutions. We have, therefore, taken concerted steps to strengthen our outreach, with particular focus on high school science educators and students. We plan to augment K–12 usage by introducing focused curricula, teacher training, and competitions such as the video challenge. In 2014, RCSB PDB coordinated development of HIV/AIDS curricula for general biology and AP biology students, in collaboration with scientists, curriculum design experts, educators, clinicians, and local high school teachers. These curricula use authentic (primary scientific) data, conform to current science teaching standards, and include hands-on teaching materials, individual and group activities, and suggestions for student assessment and curriculum evaluation. Initial activities in the curricula explain the structure and function of biological molecules playing key roles in human immunity. A separate module focuses on the targeted nature of HIV infection affecting helper T-cells and both current and emerging strategies for treating HIV infection (Fig 6). Version 1.0 of the general biology and AP biology curricula are available from PDB-101 at http://education.rcsb.org/curriculum.

## Future Plans

Since 2013, RCSB PDB educational and outreach efforts have been integrating basic science and medicine by focusing on an important public health-related topic. Our current focus is on HIV/AIDS. We use customized educational materials that showcase various HIV proteins from the PDB archive and explain at the molecular level in atomic detail how the US Food and Drug Administration (FDA)-approved combination anti-retroviral therapy (cART) regimens target selected HIV proteins. Relevant MotM features are presented using multiple modalities, including a video challenge and newly developed hands-on educational materials. Beginning in 2016, we will focus on type 2 diabetes mellitus. Going beyond the MotM feature on insulin, new educational material will be developed on the insulin receptor, insulin signaling, glucagon and its receptor, glycated hemoglobin A1c, and the mechanisms of action of glucose-lowering drugs approved by the US FDA.

Historically, some of the topics discussed in the MotM features have been selected in response to community requests. Features on catalase and carbonic anhydrase, commonly used in high school and college laboratory exercises, were both developed following educator suggestions, and are currently the third and fourth most frequently used features. Most recently, MotM features have been inspired by current events, such as the Ebola crisis in west Africa, which inspired us to produce a video on the molecular anatomy of the virus (http://www.youtube.com/watch?v=9SptF9bCyd0). The RCSB PDB welcomes requests and suggestions for new MotM features. Our overall goal in all these efforts is to bring a molecular structural view of biology and medicine to a large and diverse community.

## Abbreviations

3-D: three-dimensional
CBM MSOE: Center for BioMolecular Modeling at the Milwaukee School of Engineering
CREST: Connecting Researchers, Educators and Students
MotM: Molecule of the Month
PDB: Protein Data Bank
RCSB PDB: Research Collaboratory for Structural Bioinformatics Protein Data Bank
SMART: Students Modeling a Research Topic
wwPDB: worldwide PDB organization
